# The Cellular and Molecular Bases of Allergy, Inflammation and Tissue Fibrosis in Patients with IgG4-related Disease

**DOI:** 10.3390/ijms21145082

**Published:** 2020-07-18

**Authors:** Song-Chou Hsieh, Chieh-Yu Shen, Hsien-Tzung Liao, Ming-Han Chen, Cheng-Han Wu, Ko-Jen Li, Cheng-Shiun Lu, Yu-Min Kuo, Hung-Cheng Tsai, Chang-Youh Tsai, Chia-Li Yu

**Affiliations:** 1 Division of Rheumatology, Immunology & Allergy, National Taiwan University Hospital, National Taiwan University College of Medicine, Taipei 10002, Taiwan; hsiehsc@ntu.edu.tw (S.-C.H.); tsichhl@gmail.com (C.-Y.S.); chenghanwu@ntu.edu.tw (C.-H.W.); dtmed170@yahoo.com.tw (K.-J.L.); b89401085@ntu.edu.tw (C.-S.L.); 543goole@gmail.com (Y.-M.K.); 2 Division of Allergy, Immunology & Rheumatology, Taipei Veterans General Hospital & National Yang-Ming University, Taipei 11217, Taiwan; darryliao@yahoo.com.tw (H.-T.L.); meikankimo@yahoo.com.tw (M.-H.C.); hctsai7@vghtpe.gov.tw (H.-C.T.)

**Keywords:** IgG4-related disease, fibroinflammatory disorder, lymphoplasmacytic infiltration, storiform fibrosis, obliterative phlebitis, modified Th2 response, follicular helper T cell, CD4^+^cytotoxic T cell, Fab–arm exchange

## Abstract

IgG4-related disease (IgG4-RD) is a spectrum of complex fibroinflammatory disorder with protean manifestations mimicking malignant neoplasms, infectious or non-infectious inflammatory process. The histopathologic features of IgG4-RD include lymphoplasmacytic infiltration, storiform fibrosis and obliterative phlebitis together with increased in situ infiltration of IgG4 bearing-plasma cells which account for more than 40% of all IgG-producing B cells. IgG4-RD can also be diagnosed based on an elevated serum IgG4 level of more than 110 mg/dL (normal < 86.5 mg/mL in adult) in conjunction with protean clinical manifestations in various organs such as pancreato–hepatobiliary inflammation with/without salivary/lacrimal gland enlargement. In the present review, we briefly discuss the role of genetic predisposition, environmental factors and candidate autoantibodies in the pathogenesis of IgG4-RD. Then, we discuss in detail the immunological paradox of IgG4 antibody, the mechanism of modified Th2 response for IgG4 rather than IgE antibody production and the controversial issues in the allergic reactions of IgG4-RD. Finally, we extensively review the implications of different immune-related cells, cytokines/chemokines/growth factors and Toll-like as well as NOD-like receptors in the pathogenesis of tissue fibro-inflammatory reactions. Our proposals for the future investigations and prospective therapeutic strategies for IgG4-RD are shown in the last part.

## 1. Introduction

IgG4-related disease (IgG4-RD) is a newly defined multiorgan immune-mediated fibroinflammatory disorder with protean clinical manifestations [1,2,3,4,5,6,7,8]. In general, human immunoglobulins (IgGs) contain four subtypes, IgG1 through IgG4, of which IgG4 is the scarcest. Nevertheless, IgG4-RD is characterized by elevated serum IgG4 levels together with tissue infiltration by IgG4-secreting lymphoplasmacytic cells. The IgG4-secreting plasma cell infiltration may cause swelling, inflammation and finally fibrosis of organs mimicking malignant tumors [6,7,8,9]. The commonly involved organs include pancreas, liver, bile duct, major salivary glands, lacrimal glands, lungs, kidneys, aorta, thyroid glands, lymph nodes, retroperitoneum, prostate, pachymeninx and other tissues/organs, thus leading to a broad spectrum of protean clinical features as shown in Table 1.

It was accepted that histopathologic findings are crucial for the diagnosis of IgG4-RD. The essential pathologic characteristics including lymphoplasmacytic infiltration, storiform fibrosis and obliterative phlebitis should be confirmed in the biopsy specimens [10,11,12]. These tissue-infiltrating B lymphocytes and plasma cells are polyclonal in nature. In addition, eosinophils are commonly present in the lesions. However, no or rare neutrophilic infiltration or necrosis can be found in the tissues. Another distinctive histopathologic finding in IgG4-RD is the storiform fibrosis. This particular fibrosis appears as an irregular whorled or cartwheel-like organization of the collagen bundles in the tissue, which is presumably produced by the myofibroblasts (MFBs) after activation by profibrotic stimuli during inflammation. However, it is also accompanied scarcely by spindle cells infiltration in the affected tissues [12]. Obliterative phlebitis is characteristic of luminal obliteration by inflammatory cells and fibrosis next to a patent artery. Table 1 summarizes the wide spectrum of protean clinical manifestations and the nature of the characteristic histopathologic findings in patients with IgG4-RD. Although clinical, serologic, radiological and pathologic features are all contributory to the classification of IgG4-RD, none of them alone can fulfill the classification criteria. Therefore, the 2019 ACR-EULAR classification criteria for IgG4-RD agreed to contain both exclusion and inclusion standards [13].

## 2. Genetic Predisposition in Patients with IgG4-RD

IgG4-RD is regarded as a complex autoimmune-mediated fibroinflammatory disorder in which type 1 autoimmune pancreatitis (AIP) is the archetype. Although a number of genetic loci have been found associated with AIP, these genetic predispositions are not consistent with the actual features described more recently because no one of them can well define the disease entity. Here, we only cite the genetic studies after genome-wide association study (GWAS) has been published. In this regard, Oguchi et al. [14] by reference from GWAS, have found that *KLF*7, *FRMD*4B, *LOC*101928923 and *MPPED*2 are the susceptibility genes that trigger lacrimal and salivary gland lesions in Japanese patients with type 1 AIP.

The mammalian homologs of yeast sterile 20 (STE 20)-like kinases (MST), MST1~MST4 and YSK1, belong to serine/threonine kinases, playing crucial roles in the control of immune cell trafficking, proliferation, differentiation and apoptosis [15]. Siedel et al. [16] have found that *MST*1 locus contains CpG islands in its promoter region and suggested that the epigenetic regulation of MST1 could control immune responses. Fukuhara et al. [17] have demonstrated that decreased expression of *MST*1 due to hypermethylation in the promoter region in regulatory T cells (Treg) can lead to IgG4-related AIP.

Fibroblast growth factor-binding protein 2 (FGFBP2) secreted by cytotoxic T lymphocytes can potently bind to FGF to enhance collagen fiber synthesis by fibroblasts (FBs). The variant sequence in FGFBP2 after binding to FGF is predicted to form a distorted coil morphology of collagen fibers as found in the storiform fibrotic tissue in IgG4-RD rather than a normal helical-turn-helix fibril structure [18]. Newman et al. [19] have reported that FGFBP2 variant is highly prevalent in circulating CD4^+^cytotoxic T cells (CD4^+^Tc) in a family rather than in sporadic IgG4-RD patients.

## 3. Intestinal Dysbiosis in Animal Model and Patients with IgG4-RD

### 3.1. Induction of AIP by Persistent Exposure to Intestinal Commensal Flora Antigens in Animal Models

Haruta et al. [20] have explored the potential effect of chronic exposure (1 week ~12 months) of a mouse model to killed *E. coli* in the pathogenesis of IgG4-RD. They have concluded that a persistent stimulation of pathogen-associated molecular pattern (PAMP) activator to innate immune system may ultimately lead to the occurrence of AIP associated with salivary gland lesions which is probably through molecular mimicry. Yanagisawa et al. [21] have further identified the outer membrane protein flagellin (FliC) of the commensal flora could induce AIP-like pancreatitis with generation of high titer of anti-lactoferrin and anti-CA II antibodies in mice after repeated inoculation. These results emphasize the importance of commensal bacteria in inducing AIP via PAMP recognizing receptors. Following this way, many authors have also demonstrated that intestinal commensal microflora-released danger-associated molecular pattern (DAMP) activator can bind to Toll-like receptors (TLRs) or nucleotide-binding oligomerization domain-like receptor (NOD-like receptors, NLRs) on basophils to skew naïve T lymphocytes to Th2 responses for the development of IgG4-RD [22,23,24,25,26].

### 3.2. Intestinal Dysbiosis-mediated AIP

It is believed that gut microbiome participates in the development of host immune system. Ahuja et al. [27] have demonstrated that antimicrobial peptides secreted by pancreatic acinar cells can shape the gut microbiome crucial for intestinal innate immunity, barrier function and host survival. Mice lacking acinar Ca^2+^ channel, Orai 1, exhibit intestinal bacterial outgrowth and dysbiosis, which ultimately lead to inflammation and death. Hamada et al. [28] have conducted a comprehensive analysis of gut microbiota in patients with type 1 AIP by next generation DNA sequencing (NGS). They have found that gut microbiota profile in AIP was different from that in chronic pancreatitis (CP) in that the proportions of *Bacteroides*, *Streptococcus* and *Clostridium* species were lower in patients with AIP. Recently, Kamata et al. [29] have confirmed that activation of innate immune response by repeated administration of intestinal microflora-derived polyinosinic–polycytidylic acid could stimulate plasmacytoid dendritic cell (*p*DC) to produce IFN-α and IL-33 with eventual development of experimental AIP. Putting these data together, intestinal dysbiosis can activate *p*DC to induce type 1 experimental AIP.

Table 2 summarizes the possible genetic factors and intestinal microflora in the pathogenesis of IgG4-related AIP.

## 4. Autoantibody Diversity in Patients with IgG4-RD

Carbonic anhydrases (CAs) are extremely basic zinc metallo–enzymes with a wide phyletic distribution. The enzyme family is important for acid–base regulation and at least seven isoenzymes (CA I–CA VII) were found in mammals [30]. Inagaki et al. [31] have reported that individuals suffering from autoimmune diseases including systemic lupus erythematosus (SLE) and Sjögren’s syndrome produced a novel antibody against a 60-kDa molecule that is identified as CAs. Nishimori et al. [32] have immunized PL/j mice intradermally with human CA II in adjuvant to induce autoimmune sialoadenitis. Besides CAs, lactoferrin (LF), a red iron-binding protein, is also present mainly in the secretion of body fluid such as pancreatic juice. Many authors successively demonstrated that anti-LF and anti-CA II [33,34,35], anti-CA I and anti-CA II [33], and anti-CA IV [36] were common nonspecific autoantibodies found in patients with AIP. Whether these autoantibodies are relevant to pathogenesis of AIP needs further investigations. Recently, a number of autoantibodies have been successively discovered by authors in addition to antibodies against CAs and LF. These unique autoantibodies include anti-pancreatic secretory trypsin inhibitor-1 (PST1) [37], anti-plasminogen- binding protein (PBP) of *H. pylori* [38], anti-pancreatic trypsinogens, PRSS_1_ and PRSS_2_ [39], anti-13.1 kDa protein in systemic IgG4-related plasmacytic syndrome (SIPS) [40] and anti-amylase-α2A [41]. Anti-prohibitin [42,43], anti-galectin-3 [43,44,45], anti-annexin A_11_ [43,46], anti-laminin 511-E_8_ [43,47] and anti-monomeric C-reactive protein (anti-mCRP) have been found in acute interstitial nephritis [48]. Table 3 summarizes the autoantibodies found in the patients with IgG4-RD. However, most of these autoantibodies belong to IgG1 subclass and only a minor proportion of them are IgG4 subtype. Since IgG4 is unable to activate complements and bind to FcγR, it remains to be answered whether autoantibodies of IgG4 subclass are pathogenic or only represent an over-reactivity to undetermined antigen, which will be discussed in detail in Section 5.2.

Aoki et al. [49] have incubated serum obtained from AIP patients with normal pancreas, liver, bile duct and salivary gland. They have found these serum samples containing IgG4 subclass antibodies that could bind to the epithelial cells from these organs. Furthermore, Shiokawa et al. [50] have injected circulating IgGs from IgG4-RD subjects into neonatal male BALB/c mice to examine their pathogenic activity. They concluded that both IgG1 and IgG4 from IgG4-RD exhibited pathogenic activity via binding to the affected tissue in this mouse model.

## 5. Development of IgG4 Antibodies by Modified Th2 Response

The production of immunoglobulins (Igs) is carried out by B cells in the presence of helper T cells. The IgG class is more complex in structure and biology than we think. The synthesis of IgG1, IgG2b, IgG3, IgG4 and IgE requires the help from Th2 cells whereas IgG2a synthesis is dependent on Th1 help. The most abundant IgG1 level in serum ranges from 5–10 mg/mL whereas the least abundant subclass IgG4 ranges from 0.35–0.51 mg/mL. However, in case of IgG4-RD, the serum levels of IgG4 may elevate to higher than 130 mg/mL. Aalberse et al. [51] have demonstrated that IgG4 antibodies commonly arise after long-term exposure to an antigen by a modified Th2 response such as in a scenario of allergen desensitization therapy. Thereby, the production of IgG4 antibody can reduce the degree of chronic allergic or inflammatory reaction to environmental stimuli by displacing the binding of IgG1 or IgE antibodies with their cognate antigens or allergens. Another two important unique properties of IgG4 antibodies are low binding affinity to C1q and FcγR on immune cells. These two unique immunological properties may originate from “Fab–arm exchange” between two IgG4 molecules to become asymmetrical antibodies with two different antigen-binding sites called as “bi-specific monovalent antibody for a given antigen” (Please see the next section in detail.)

### 5.1. Modified Th2 Response for the Class-switch from IgE to IgG4

It is well established that there are strong links between IgG4 and IgE. Usually, IgG4 responses are connected with IgE-mediated allergic reaction since both antibodies are induced by Th2 cytokines, mainly IL-4 and IL-13 [52]. However, the two cytokine receptors are distinct since production of IgE antibodies often occurs before IgG4 [53]. Thus, a ‘modified Th2 response’ is defined as “presence of IgG4 antibodies in the absence of IgE antibodies” [54]. Aalbese et al. [55] have further reported that DNA encoding IgG4 is upstream to the DNA encoding IgE and is deleted during the class switch to IgE in normal situation. However, Jeanine et al. [56] have demonstrated that the key cytokine driving “reverse” IgE/IgG4 class switch in the modified Th2 response is IL-10 which promotes IgG4 production but inhibit IgE production.

Recent investigations by van der Neut Kolfschoten et al. [57] have demonstrated that in the context of an allergic response, IgE-producing plasma cells require not only IL-4, but IL-5, IL-6, IL-7, IL-9 and IL-13 to help synthesize IgE. In addition to Th2 cytokines, T follicular helper 2 (T_fh2_) cell-derived cytokine, IL-21, is also involved in determining IgG4/IgE ratio [58,59]. Recently, Akiyama et al. [60] have confirmed that IgG4/IgG ratio was statistically higher in the presence of IL-4 alone than in the presence of T_fh2_ cytokines including IL-21 alone in patients with IgG4-RD, but not in healthy individuals. In brief conclusion, IL-10 is implicated in the class switch from IgE to IgG4 production. Furthermore, IL-10 is needed for driving the differentiation of IgG4-class-switched B cells to IgG4-secreting plasma cells. IL-21 also exerts a similar effect with IL-10 and is one of the major cytokines that drive IgG4 shift. 

### 5.2. Odd Immunological Properties of IgG4 Antibody

#### 5.2.1. Fab–arm Exchange Between 2 Different IgG4 Antibodies Resulting in Non-inflammatory Properties of IgG4 Antibody

Van der Zee et al. [61,62] are the 1st researcher to note that human IgG4 antibodies against allergen is unable to link across the identical antigens, in which functional change to monovalency of IgG4 antibodies is suspected. This unique immunological property can be applied in the allergen-induced immunotherapy (AIT) and the production of IgG4-therapeutic antibodies in clinical practice [63,64,65,66]. Aalberse et al. [67] have compared the CH3 domain of IgG1 and IgG4 and found 3 amino acid differences at glutamine (Q)355 in IgG4 *vs.* arginine (R)355 in IgG1, (R)409 in IgG4 *vs.* lysine (K)409 in IgG1 and L445 in IgG4 *vs.* P445 in IgG1. Because residue 409 is located in the interface between the two CH3 domains, the mutation (K→R) may affect the stability of the non-covalent interaction between H-chains. In contrast, Schuurman et al. [68] observed that cross-linking of two non-identical antigens by IgG4 antibodies may occur via bi-specificity of IgG4 antibodies. In exploring the molecular basis of IgG4 Fab–arm exchange in the core sequence of IgG4 hinge region (residues 226–230), Aalberse group [69,70] have found a replacement of serine (S) for proline (P) in this interval promotes formation of intra- rather than inter-H chain disulfide bonds. However, the dissociation of the CH3 domains seems to be a rate-limiting step in the Fab–arm exchange process [51,70,71,72,73]. The “Fab–arm exchange” property of IgG4 antibody leads to the formation of monovalent bi-specific antibody, a poor interaction with FcγRII and/or FcγRIII, an absence of complement C1q binding activity, the formation of small non-precipitating immune complexes and an eligibility for the production of therapeutic antibodies [66,67]. We have summarized the differences in the regulation, mode of action and clinical application between IgE & IgG4 antibodies in Table 4.

#### 5.2.2. A Unique Conformation of FG Loop in the CH2 Domain of IgG4 Molecule

Davies et al. [69,71], by using high-resolution crystal structure analysis at 1.9 Å and 2.35 Å, have revealed a unique conformation for the FG loop structure in the CH2 domain of IgG4 different from that of the IgG1 molecule. This loop could explain the poor FcγRII, FcγRIII and C1q binding capacities of IgG4 compared to IgG1 and IgG3 via preclusion of any interaction of immune complexes with the lower hinge region of IgG4 backbone and subsequent facilitation of Fab–arm exchange between two IgG4 antibody molecules [66,67]. Moreover, this CH2 domain in the FG loop also alters conformingly the C1q binding site [74].

#### 5.2.3. Rheumatoid Factor-like Fc Binding Activity of IgG4 in the Autoimmune and Inflammatory Pathology

Contradictory to anti-inflammatory and anti-allergic activity, the IgG4 molecule *per se* can undergo Fc-mediated aggregation via the binding site in IgG4-CH2 and neighboring IgG4-CH3 interface to form aggregated IgG4 similar to IgG-rheumatoid factors [75]. Different from the conventional RF, which binds via its variable F_(ab’)2_ domains, the activity of IgG4-“RF” relies on its constant Fc domain [51,75].

#### 5.2.4. Pathologic Roles of IgG4-autoantibodies in Certain Autoimmune Diseases

It is well-known that most of the antibody-mediated autoimmune diseases are caused by IgG1 and IgG3 autoantibodies. However, Rock et al. [76] have noted that pemphigus vulgaris, a skin blister disease, is hallmarked by IgG4-autoantibodies in the affected skin tissue. To date, at least 13 IgG4-related autoimmune diseases have been reported including myasthenia gravis with muscle-specific kinase IgG4 antibody and idiopathic membranous nephropathy with IgG4 anti-phospholipase A2 receptor [77,78,79]. These aberrations are compatible with the observations by Aoki et al. [49] that IgG4 obtained from patients with IgG4-RD bound to normal epithelial cells and exerted pathologic effects on different tissues, which are quite different from the protective effect of IgG4 antibody to damp harmful effects of immune complexes in classical autoimmune-mediated tissue damage [51,63,66,67].

#### 5.2.5. The Glycosylation Patterns of IgG4 Molecule Induce Complement Activation in Some IgG4-RD Patients with Hypocomplementemia and Primary Sclerosing Cholangitis

Usually, the IgG4 molecule exhibits low C1q binding capacity and does not activate classical complement system as stated in Section 5.2.1. However, 40–50% of patients with IgG4-related renal inflammatory diseases are found to have soluble immune complex formation and hypocomplementemia with low C2, C4 levels and CH50 activity in the serum [80]. Nuraki et al. [81] have reported that 36% of AIP patients are with low C3 and C4 levels and 17% of them have low CH50. Moreover, Deshpande et al. [82] and Cornell et al. [83] have observed that immune complex (IC) formation is playing a role in the pathophysiology of IgG4-related AIP patients. Sugimoto et al. [84] have analyzed the PEG-precipitated IC from IgG4-RD patients and found that IgG4 could participate in the activation of complements in IgG4-RD patients with hypocomplementemia. To elucidate the inconsistency in complement-activating effect of IgG4 antibody, Konno et al. [85] have analyzed the N-linked glycan of IgG4 molecule derived from patients with IgG4-RD. They found decreased galactosylation of IgGs is irrelevant to complement activation whereas IgG4 fucosylation may lead to complement activation, hypocomplementemia and various organ damages in patients with IgG4-RD. Furthermore, Culver et al. [86] have shown that an increase in IgG4-specific Fc fucosylation and hybrid structures in IgG2/3 Fc portions that are proinflammatory in nature are found in IgG4-RD and primary sclerosing cholangitis.

Putting these results together, IgG4 antibodies with particular N-glycan alterations can activate complements and are associated with hypocomplementemia in some IgG4-RD patients. We have summarized the odd immunological properties of IgG4 antibodies in patients with IgG4-RD in Table 5.

## 6. Eosinophilia, Hyper-IgE Levels and Allergy in Patients with IgG4-RD

It is well known that IgG4 levels rise after IgE concentration declines in the hyposensitization therapy in allergic disorders. Scientists have thus hypothesized that IgG4 perhaps emerges to shutdown allergen offense in an attempt to attenuate allergic inflammation elicited by noxious environmental allergens. It has also long been recognized that a proportion of patients with type 1 AIP have histories of allergies, peripheral eosinophilia and elevated serum IgE or manifestations of atopy during the development of AIP [87]. Van Toorenenbergen et al. [88] have demonstrated a significant correlation between total IgE and total IgG4 levels in patients with AIP and patients with atopic diseases, but not with patients with pancreatic cancer. The group has also found that the serum IgE/IgG4 ratio in patients with atopic diseases is significantly different from the ratio in patients with AIP. For exploring the roles of IgE and allergic diseases in the diagnosis as well as pathogenesis of AIP, Zhang et al. [89] have surveyed the patients with AIP. They discovered that half of their Chinese AIP patients suffered from allergic conditions whether they have high or low serum IgE levels. Those with higher IgE levels and allergic conditions suffered from more seasonal changes in pancreatic inflammation with increased mast cell number. They concluded that allergic processes may play an important role in the AIP pathogenesis. Della-Torre et al. [90] have also concluded that the processes inherent to IgG4-RD itself rather than atopy *per se* may contribute to eosinophilia and hyper-IgE without atopy. To solve the enigma regarding allergic reactions, eosinophilia and hyper-IgE levels in IgG4-RD, some authors have revealed that IL-10 can suppress allergen-specific accessory function of monocytes and Th2 responses, thereby decrease the IL-4-induced IgE synthesis [91,92,93]. As shown in Table 5, IL-10 is essential for cross-switch of IgE to IgG4 and is also crucial for IgG4 secretion by B cells. Accordingly, atopic reaction during the processes of AIP development may stimulate eosinophilia &/or IgE production, but irrelevant to allergic reaction in 40-50% of AIP patients. Demographic study conducted by Saeki et al. [94] has shown that younger age onset, female preponderance and upper-body organ involvement are characteristic in 43% of IgG4-RD patients with allergic condition. In clinical practice, Culver et al. [95] have reported high serum IgG4 levels in 81%, and high serum IgE levels in 54% of patients with IgG4-RD. Moreover, an IgE-mediated allergic response tends to develop in most of the IgG4-RD subjects. Thus, the serum IgE level could be used in the diagnosis and prediction of atopy relapse in these patients. On the other hand, whether the AIT by repeating injections of low-dose allergens to induce IgG4 class-switching from IgE to IgG4 can result in IgG4-RD remains in debate. Della-Torre et al. [96] analyzed 116 patients with biopsy-proven IgG4-RD to link AIT and IgG4-RD. Their results did not support obvious association between the two events although an increased tendency of high IgG4 in AIT group was observed. We speculate that elevated serum IgE levels is responsible for allergen-specific allergic reactions whereas serum IgG4 elevation is not the necessary parameter for the occurrence of IgG4-RD unless changes of glycosylation pattern in IgG4 molecules have occurred as stated in Section 5.2.3.

## 7. The Pathogenic Role of B Cells in Chronic Inflammation and Storiform Fibrosis in Patients with IgG4-RD

### 7.1. The Pathogenic Roles of CD19^+^ Plasmablasts in AIP Patients

Until now, the actual pathogenesis of IgG4-RD has remained elusive. The characteristic fibroinflammatory tissue in IgG4-RD contains many IgG4-producing plasmacytes/plasmablasts embedded in a fibrotic matrix that are originating from activated mesenchymal cells. New insights into cross-talk among different immune-related cells including innate immune cells, T cell subsets, B cell subset and fibroblasts/myofibroblasts have been discussed by Touzani et al. [97]. Recent studies have documented heterogeneity of the functions of B cell lineages, including cytokine secretion, antigen presentation, autoantibody production and modulation of T-B interactions, which would contribute to tolerance, autoimmunity as well as autoimmune diseases. In the tissue specimens of IgG4-RD, ectopic germinal center formation with dense infiltration by lymphoplasmacytes is prominent in salivary glands, which is probably caused by IL-21 [98]. Besides, the de novo oligoclonal expansion of circulating CD19^+^ plasmablasts in active and relapsing IgG4-RD has been observed, which could be used as a diagnostic biomarker for IgG4-RD independent of serum IgG4 [99]. The CD19^+^plasmablasts may produce proinflammatory cytokine (IL-1), profibrotic factors (TGF-β and platelet derived growth factor B [PDGFB]) and a diverse autoantibodies (as shown in Table 3) in mediating tissue inflammation, destruction and fibrosis. Accordingly, anti-CD20 monoclonal antibody (rituximab) appears to be an effective treatment for IgG4-RD, even without combination of glucocorticoids in clinical practice [100]. On the other hand, regulatory B cell (Breg) mainly originating from the marginal zone of lymph nodes can produce IL-10 and TGF- β for regulatory T cell (Treg) differentiation and Th1/Th2 polarization [101]. Sumimoto et al. [102] and Lin et al. [103] have specifically demonstrated that in addition to plasmacytes, there are two distinct Breg subsets, CD19^+^CD24^high^CD27^+^ Breg, which activates.and CD19^+^CD24^high^CD38^high^ Breg, which suppresses the activity of AIP (please see more details in Section 8.3). Another B cell subpopulation, CD27+ memory B cell can express higher affinity cell receptor as potent APC to produce high affinity antibodies particularly in the later phases of chronic immune reaction [104]. The glucocorticoid treatment for IgG4-RD can reduce naïve B cell, circulatory plasmablasts/plasma cells and increase memory B cells [105].

Another histopathologic hallmark of IgG4-RD is the presence of storiform fibrosis in some of the affected tissues characterized by a dense tissue fibrosis with an irregular whorled organization of the collagen bundles. However, renal fibrosis is relatively rare, and interstitial nephritis as well as membranous glomerulonephritis are more frequent in IgG4-related nephropathy. On the other hand, nodal fibrosis is relatively rare, but granuloma formation is frequent in IgG4-related lymphadenopathy. It is conceivable that the histopathologic features would inevitably cause lack or scarcity of fibrosis in these two IgG4-related illness. Fukui et al. [106] have observed through transmission electron microscopy an abnormal periodicity of collagen bundles and epithelial–mesenchymal transition (EMT) of FBs to MFBs without reducing E-cadherin expression. These may lead to increases in filamentous actin and α -smooth muscle actin, SNAIL, as well as heat shock protein 47 found in storiform fibrosis of patients with IgG4-RD.

### 7.2. The Ontogenesis of MFBs

It is believed that chronic inflammation can accelerate the progression of fibrosis in IgG4-RD. MFBs are the major effector cells of tissue fibrosis arising from FBs, pericytes, epithelium, endothelium or smooth muscle cells. After activation by fibrosis-related inflammatory mediators, the mesenchymal cells begin to trans-differentiate to MFB, proliferate and produce an excessive amount of extracellular matrix including fibrillary collagens (collagen I, III), glycoproteins (fibronectin, fibrillin, elastin and proteoglycans) and non-fibrillary collagen (collagen IV) that can deposit in the tissue [107].

### 7.3. The Fibrosis-related Inflammatory Mediators

Recently, a bunch of fibrosis-related inflammatory mediators have been successively discovered. These fibro-inflammatory mediators include IL-1 family (IL-1α, IL-1β, IL-18, IL-33, IL-36 α, IL-36β and IL-36γ) [107], Th1 cytokine (IFN- γ) [108], Th2 cytokines (IL-4, IL-5, IL-10 and IL-13), Th17 cytokines (IL-17 and IL-22), innate immune cell-derived proinflammatory cytokines (IL-6 and TNF- α), growth factors (TGF-β, PDGF, CTGF, IGF, FGF, EGF and VEGF) and chemokines (CCL2, CCL3, CCL4, CCL20) [109]. Furthermore, Kotsiou et al. [110] have documented that IL-33/ST2 (suppression of tumorigenicity 2) axis can facilitate EMT process of various cell types and abnormal FB proliferation, ultimately leading to tissue fibrosis. Kawashiri et al. [111] have concluded that increased growth differentiation factor 15 (GDF-15), as a serologic surrogate marker for fibrosis-related processes, may precisely reflect the degree of tissue fibrosis in patients with IgG4-RD.

### 7.4. Pathogenic Roles of B Cell Subsets, B Cell-derived Factors and Help Signals in the Tissue Fibrosis of Patients with IgG4-RD

**Figure 1:**
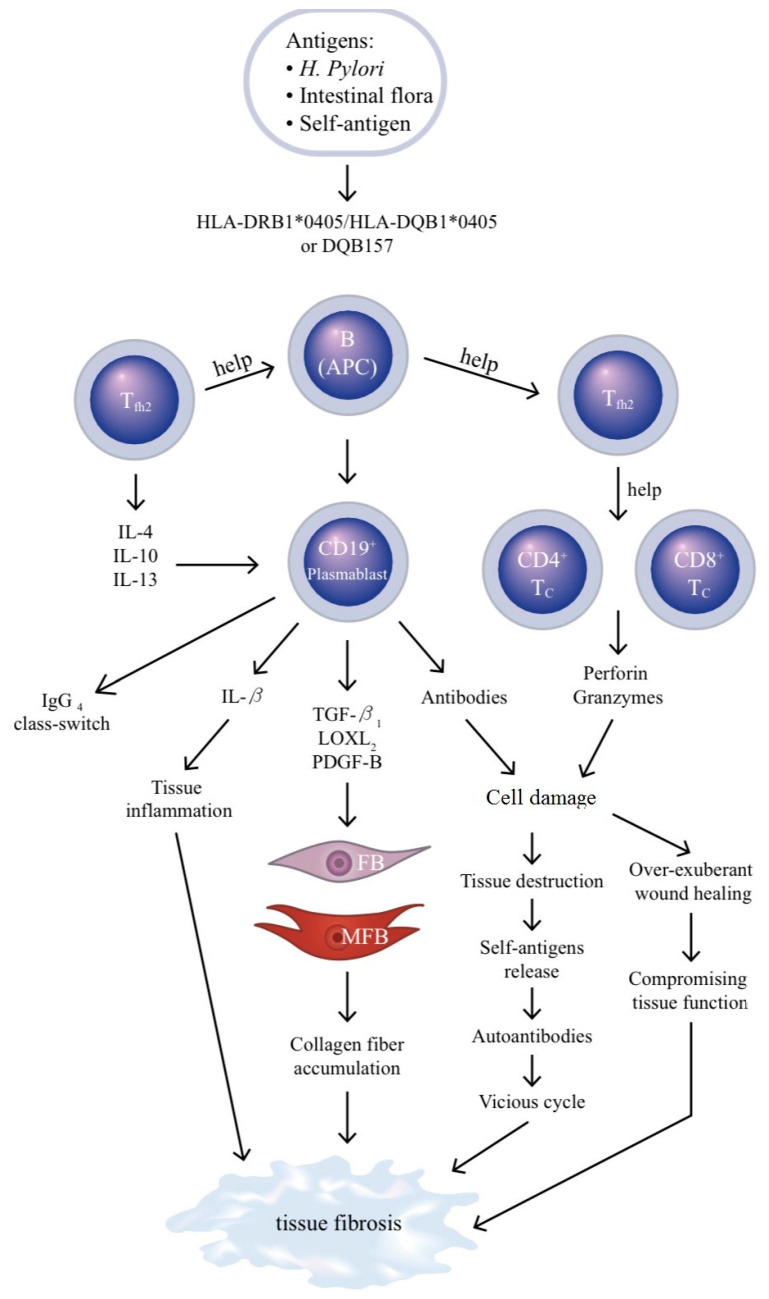
Pathogenic role of the B lymphocyte as antigen-presenting cell (APC) and effector cell in the tissue fibrosis of patients with IgG4-related disease. The genetically predisposed B cells (acting as APCs) bind to environmental offending agents (e.g., *H. pylori*), intestinal microflora or autoantigens and then provide signals to CD19^+^ plasmablasts to mediate effector functions including: (1) IgG4 class-switch with the help of follicular helper T2 (T_fh2_), (2) IL-1β production to cause tissue inflammation, (3) production of profibrotic molecules (TGF-β, LOXL2, PDGF-B) to activate fibroblasts (FBs) and myofibroblasts (MFBs) in charge of collagen fiber production and deposition in the tissue and (4) autoantibody production to contribute to tissue destruction. Moreover, B cells help T_fh2_ cells facilitate the ontogenetic differentiation of the two cytotoxic T cells (Tc), CD4^+^Tc and CD8^+^Tc, in tissue destruction. LOXL2—lysyl oxidase-like 2; PDGF—platelet derived growth factor. For abbreviations not designated here, please see the abbreviation list in the main text.

It is postulated that B lymphocytes and their lineages, Breg’s, participate in tissue fibrosis of IgG4-RD. In an in vitro study, Francois et al. [112], by co-culturing peripheral blood B cells and dermal FBs isolated from systemic sclerosis patients, have found an increase in IL-6, TGF-β1, CCL2, collagen synthesis, α-SMA, TIMP and MMP-9 in dermal FBs. This enhancement can be further augmented by the addition of B cell-activating factor of TNF family (BAFF). Della-Torre et al. [113] have proved that B cell depletion by therapeutic antibody attenuates serologic biomarkers of fibrosis and MFB activation in patients with IgG4-RD. The same group has further directly demonstrated that B cells obtained from IgG4-RD patients could produce many different profibrotic molecules including PDGFB for stimulating collagen production from FBs, lysyl oxidase like 2 (LOXL2) for extracellular matrix remodeling and various chemotactic factors (CCL4, CCL5, CCL11) for chemo-attraction and activation of FBs [114]. Puente et al. [115] have even proposed that LOXL2 may become a new target for anti-fibrogenic therapy. Breg, via secretion of regulatory cytokines such as IL-10 and TGF-β, can contribute to fibrosis [101,102,103]. It is particularly interesting that the antigen-presenting function of memory B cells can enhance proliferation and maturation of follicular helper T_2_ cell (T_fh2_) to become CD4^+^- and CD8^+^-cytotoxic T cell (Tc) populations [104]. The two cytotoxic T cell subpopulations then secrete perforin and granzymes (A and B) to induce apoptosis of non-immune, non-endothelium mesenchymal cells, inflammation and finally fibrosis in IgG4-RD [116]. On the other hand, after receiving help signals from T_fh2_ cells, B cells mature to CD19^+^ plasmablasts, which can subsequently produce autoantibodies, proinflammatory cytokine (IL-1β) and profibrotic cytokines (TGF-β1, LOXL2 and PDGFB) to enhance collagen fiber synthesis and deposition driven by FBs and MFBs. All of these B cell-derived factors and help signals hasten tissue inflammation and consequently tissue fibrosis in patients with IgG4-RD. Figure 1 depicts the pathogenic role of B lymphocytes as APC and effector cells in the tissue inflammation and fibrosis of patients with IgG4-related disease.

## 8. The Cellular and Molecular Bases of Allergy, Inflammation and Tissue Fibrosis in Patients with IgG4-RD

In addition to the crucial roles of B cell subpopulations as antigen-presenting and effector cells in the pathogenesis of tissue fibrosis in patients with IgG4-RD, recent investigations have further revealed that many innate immune cells including white blood cell lineages (neutrophils, basophils and eosinophils), and macrophage type 2 (M2–M*φ*)/plasmacytoid dendritic cells (*p*DC) [117,118] and different T cell subsets including, Th2, Treg, T_fh2_ and CD4^+^ - and CD8^+^-cytotoxic T cells (CD4^+^and CD8^+^ Tc) play active and critical roles in the immunopathogenesis of IgG4-RD [101].

### 8.1. Involvement of Innate Immune Cells in Patients with IgG4-RD

Triggering of innate immune responses by microbe-associated molecular patterns (MAMPs) and damage-associated molecular patterns (DAMPs) is mediated by binding of these molecules to the TLRs and NOD-like receptors (NLRs) on innate immune cells including M*φ*_2_/*p*DC, basophils and polymorphonuclear cells (PMN). The binding leads to release of proinflammatory cytokines (IL-1 and IL-6), B cell growth factors (BAFF and APRIL) and Th2 differentiation cytokine (IL-4) to induce B cell maturation and Igs class-switch from IgE to IgG4 by modified Th2 response. For clarifying the properties of fibroinflammatory cytokines and their individual producing cells, many authors have successively demonstrated that IL-10 and IL-13 released from Th2, B lymphocyte-activating factor of TN**F** family (BAFF) and a proliferating inducing ligand (APRIL) released from M2-M*φ* and PMN, IL-4 and IRF-7 released from *p*DC, IL-33 released from antigen presenting cell (APC) and endothelial cell (EC) in patients with IgG4-RD [117,118,119,120,121,122]. In addition, activated M2-M*φ* may release profibrotic cytokines (TGF-β and IL-33) to activate the production of profibrotic cytokines from Treg including TGF-β, IL-10 and IL-33. These effects can further stimulate FBs and MFBs to synthesize collagen fibers for storiform fibrosis in IgG4-RD.

### 8.2. The Immunopathologic Roles of Aberrant Functions of Treg, T_fh_ and CD4^+^ and CD8^+^Tc Subsets in Patients with IgG4-RD

Recent studies have revealed that increased proportions of Th2 and Treg cells, with their abnormal cytokine production, are involved in IgG4-RD and CD4^+^T cell infiltration constitutes the major inflammatory cell populations in IgG4-RD lesions. Tsuboi et al. [123] have detected the mRNA expression of Treg and Th2 cytokines in PBMCs and labial salivary glands by RT-PCR. They have concluded that overexpression of IL-10, TGF-β and activation-induced cytidine deaminase (AID) in salivary glands are involved in the pathogenesis of IgG4-RD. Generally, Treg cells are usually subdivided into 2 groups: (1) naturally occurring Treg, with CD45RO^+^CD25^high^CD4^+^ surface marker and (2) naïve Treg with CD45RA^+^CD25^+^CD4^+^ surface marker. Miyoshi et al. [124] have reported that increased numbers of naturally occurring Treg in the circulation can affect IgG4 production in AIP whereas decreased numbers of naïve Treg may be implicated in the pathogenesis of AIP.

Histologically, ectopic germinal center (GC) formation is present in some lesional tissues of patients with IgG4-RD. Wurster et al. [125] have documented that IL-21 is crucial for Th2 cytokine productions and can specifically inhibit the differentiation of naïve helper T cells to INF-γ producing Th1 cells. Furthermore, IL-21 production by T_fh_ that expresses CXCR5 (corresponding ligand is CXCL13) chemokine receptor can help GC formation [98]. T_fh_ cells are a distinct CD4^+^T cell subpopulation that can expedite B and plasma cell differentiation and finally GC formation. Aberrant expansion and function of T_fh_ subsets can be found associated with circulating plasmablast numbers, IgG4, IL-4 levels and tissue damage in patients with IgG4-RD [126,127]. Further investigations by Grados et al. [128] have revealed that IgG4-RD patients showed an increase in circulating Treg, Th17 and CD4^+^CXCR5^+^PD1^+^T_fh_ cell populations. The increased T_fh_ cells were caused by a specific expansion of T_fh2_ (CCR6^-^CXCR3^-^), and to a lesser extent, T_fh17_ (CCR6^+^CXCR3^-^) subsets. Therefore, IgG4-RD is characterized by a shift of circulating T cells toward a Th2/T_fh2_ and Th17/T_fh17_ polarization.

Although CD4^+^T helper cells are regarded as the most abundant cells in the GC of affected tissues in IgG4-RD, these cells are considered as a driver in the pathogenesis of the disease. Another unique CD4^+^T cells existing in lesional sites are CD4^+^ cytotoxic T cells (CD4^+^Tc) which secrete cytolytic molecules including perforin and granzymes A and B. Moreover, profibrotic cytokines (IL-1β, TGF- β) and Th1 cytokine (IFN- γ) are also secreted by these cells [129,130,131]. Some of these unique cells also bear signaling lymphocytic activation molecule F7 (SLAMF7) surface marker which is usually presented on the plasmablasts and plasma cells, but not CD4^+^T cells [132]. Della-Torre et al. [133] have identified a subset of CD4^+^SLAMF^+^CD8a^-^ T_EM_ (CD45RO), the cytotoxic T cells with effector memory phenotype, which is oligoclonally expanded in patients with active IgG4-RD and can be suppressed following glucocorticoid administration to remit disease. In short conclusion, both CD4^+^CTL and CD8^+^CTL in disease lesions of IgG4-RD may contribute to the induction of cell apoptosis and tissue fibrosis of the disease [116].

### 8.3. Involvement of Abnormally Functioning Bre FliC g cells in Patients with IgG4-RD

**Figure 2:**
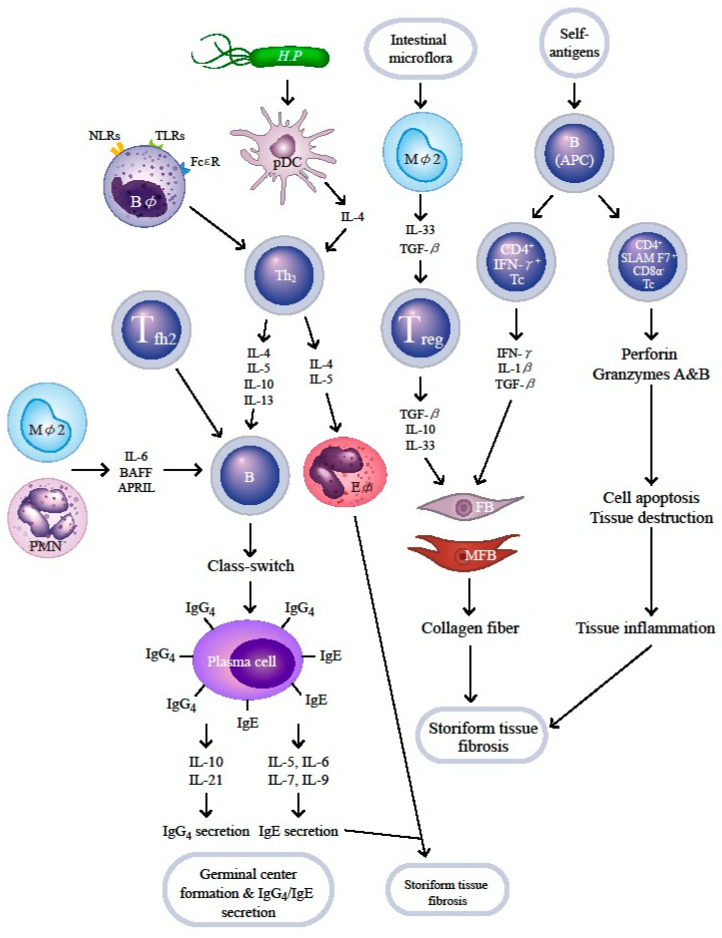
Cellular and molecular bases of immunopathogenesis in patients with IgG4-related disease. The environmental or self-antigens such as PAMP or MAMP bind to the TLRs/NLRs on basophils (B*φ*) and plasmacytoid dendritic cells (*p*DC), skewing naïve T cells to Th2 subset. Upon the effects of Treg, T_fh2_, M2 macrophage (M*φ*2,) and PMN, the B cells mature and undergo Ig class-switch from IgE to IgG4 by the modified Th2 response in the ectopic germinal center of fibroinflammatory tissue. The IL-4- and IL-5-stimulated eosinophils (E*φ*) and the residual IgE mediate allergic reaction. On the other hand, B cells (as APC), M*φ*2, and Treg provide profibrotic cytokines to facilitate production and accumulation of collagen fibers by fibroblasts (FBs)/myofibroblasts (MFBs) to mediate storiform fibrosis. Furthermore, B cells as APCs enhance generation of two populations of CD4^+^cytotoxic T cells (CD4^+^Tc), CD4^+^IFN-γ^+^Tc and CD4^+^SLAMF7^+^CD8α^-^Tc, to induce cell apoptosis and tissue destruction by the secreted perforin and granzymes (A and B). TLR—Toll-like receptor; NLR—nucleotide-binding oligomerization domain like receptor; Th2—helper T cell type 2; BAFF—B cell-activating factor of tumor necrosis factor family; APRIL—a proliferation-inducing ligand of B cell. For the abbreviations not designated here, please see the abbreviation list in the main text.

Kessel et al. [134] have found an antigen-driven B cell with CD19^+^CD25^high^ surface marker which expresses a regulatory effect to suppress proliferation of autoreactive T lymphocytes. IL-10 is believed to be the major effector molecule released from these Breg cells to exert immune-regulatory functions. Authors have also demonstrated that IL-10 is produced by both CD19^+^CD24^high^CD27^+^ (B10) and CD19^+^CD24^high^CD38^high^ (immature) Breg subsets. Sumimoto et al. [102] and Lin et al. [103] have demonstrated that these two Breg cell aberrations, increased immature Breg subset, but decreased B10 type Breg subset, are involved in IgG4-RD. However, more studies are required to get a definite conclusion. In conjunction with the context of Section 7, we provide a comprehensive scheme to illustrate the cellular and molecular bases of allergy, chronic inflammation, and storiform tissue fibrosis in patients with IgG4-RD in Figure 2.

## 9. Conclusions

IgG4-RD is a complex, protean and multiorgan fibroinflammatory disease originated from overactive IgG4-producing lymphoplasmacytic cell infiltration in variable tissues. It may thus lead to bizarre clinical and histological manifestations including storiform tissue fibrosis and obliterative phlebitis. The odd properties of IgG4 molecules such as Fab–arm exchange, Fc–Fc aggregation and modified Th2 response lead to immunological paradox in IgG4-RD. A series of florid interactions among innate and adaptive immune cells result in abnormal cytokine/chemokine/growth factor modulation that may lead to fibroinflammatory cell infiltration, tissue inflammation with fibrosis and allergic conditions in patients with IgG4-RD. Many conundrums remain to be solved such as the nature of antigenic stimuli, the molecular mechanism underlying storiform fibrosis, the etiology of aberrant immunobiology, the fine-tuning of genetic/epigenetic modifications of cytokine expression, and the pathologic roles of autoantibodies relevant to immunopathogenesis of IgG4-RD.

## 10. Future Prospects

With regards to the designation of new therapeutic strategies for IgG4-RD other than corticosteroids, nonspecific immunosuppressants and anti-CD20 therapy are mandatory in clinical practice. These strategies may include blockades of modified Th2 response and new anti-fibrogenic therapies. However, many unmet needs should be fulfilled for identifying more in-depth the cellular and molecular mechanisms underlying IgG4-RD—as well as improving its therapies. These investigations may focus on:

1. The precise epigenetic regulations, including DNA methylation/acetylation and histone modifications for the diverse immune dysfunctions in IgG4-RD;
2. The aberrant expression of non-coding RNAs in the ontogenesis of abnormal B cell biology in IgG4-RD;
3. The characterization of factors involved in the induction of CD^4+^Tc in IgG4-RD;
4. The elucidation of the sophisticated molecular mechanism underlying storiform fibrosis;
5. Clarification of the interactions between *H. pylori* infection and other environmental factors, such as allergens for development of the disease;
6. Immunopathologic roles of different IgG4 autoantibodies in its pathogenesis;

These are only a few points we suggest that may be helpful for understanding IgG4-RD, as well as the unique nature of IgG4, *per se*. Since the disease is a relatively newly found disorder implicating the humoral immune system, a multi-arrays of work are expected to have fruitful results in the future.

## Figures and Tables

**Table 1:** A wide range of protean clinical manifestations and characteristic histopathologic findings in patients with IgG4-related disease.

**(1) Clinical manifestations** [1,2,3,4,5,6,7,8]	
Type 1 autoimmune pancreatitis	IgG4-related pachymeningitis
IgG4-related dacryoadenitis	IgG4-related hypophysitis
IgG4-related sialoadenitis	IgG4-related aortitis/periaortitis/arteritis /mediastinitis/mesenteritis
Küttner’s tumor (submandibular sialodenitis)	IgG4-related pleuritis/pericarditis
Mikulicz’s disease (sialoadenitis +dacryoadenitis)	InG4-related mastitis
IgG4-related orbital myositis	Ormond’s disease (retroperitoneal fibrosis)
Riedel’s thyroiditis	IgG4-related membranous glomerulonephritis
IgG4-related allergic rhinitis	IgG4-related ureteritis/urethritis
IgG4-related asthma	IgG4-related prostatitis
IgG4-related chronic rhinosinusitis	IgG4-related skin diseases
IgG4-related lung disease / pseudotumor	IgG4-related lymphadenopathy IgG4-related midline destruction lesion
IgG4-related sclerosing cholangitis	
IgG4-related cholecystitis	
IgG4-related hepatitis	
**(2) Characteristic histopathologicalfeatures** [9,10,11,12]	
Lymphoplasmacytic infiltration: IgG4 (+) plasma cell/IgG (+) plasma cell ratio >40%	
Storiform fibrosis: irregular whorled organization of the collagen bundles throughout the tissue led by the activation of myofibroblasts after profibrotic stimuli of inflammation	
Eosinophil, but not neutrophil infiltration, is commonly present Absence of granuloma or tissue necrosis	
Obliterative phlebitis: partial or complete obliteration of medium-sized veins by lymphoplasmacytic cell infiltration appearing as an inflammatory nodule next to a patent artery	

**Table 2:** Genetic loci and intestinal microflora involved in the pathogenesis of IgG4-related autoimmune pancreatitis (AIP).

**(1) Genetic loci**
*KLF*7, *FRMD4B*, *LOC101928923*, MPPED2 in Japanese AIP associated with lacrimal/salivary gland lesions [14] Decreased *MST_1_* of regulatory T in Japanese AIP with extra-pancreatic lesions [17]
*FGFB*P2 (fibroblast growth factor binding protein type 2): single base deletion in IgG4-RD [19]
**(2) Persistent exposure of intestinal commensal flora antigen in mouse AIP model**
Avirulent *E. coli* (as PAMP activator) induces anti-CA II, anti-LF and ANA in mouse AIP with salivary gland involvement [20]
Commensal *E. coli*-derived membrane protein flagellin (FliC) induces AIP-like inflammation in mouse model [21]
Intestinal microflora can activate TLRs and NLRs on basophils to promote Th2 skewing and IgG4 production in the presence of BAFF [22,23,24,25,26]
**(3) Intestinal dysbiosis-mediated AIP development via *p*DC activation**
Decrease in gut *Bacteroides*, *Streptococcus* and *Clostridium* species in patients with AIP, compared to chronic pancreatitis [28]
Activation of *p*DC by innate immune responses against intestinal dysbiosis in experimental mouse AIP [29]

BAFF—B cell-activating factor of TNF family; *p*DC—plasmacytoid dendritic cell; CA—carbonic anhydrase; LF—lactoferrin; ANA—antinuclear antibodies.

**Table 3:** The presence of autoantibodies in patients with IgG4-related disease.

Anti-carbonic anhydrase II [33,34,35]
Anti-carbonic anhydrase I [33] and IV [36]
Anti-pancreatic secretary trypsin inhibitor-1 (PST1) [37]
Anti-plasminogen-binding protein (PBP) of *H. pylori* [38]
Anti-pancreatic trypsinogens PRSS1 and PRSS2 [39]
Anti-13.1 kDa protein in systemic IgG4-related plasmacytic syndrome (SIPS) [40]
Anti-amylase-2A [41]
Anti-prohibitin [42,43]
Anti-galectin-3 [43,45]
Anti-annexin A11 [43,46]
Anti-laminin 511-E8 [43,47]
Anti-monomeric C-reactive protein (mCRP) in acute interstitial nephritis [48]

**Table 4:** Differences in the regulation, modes of action and clinical applications between IgE and IgG4 antibodies.

Parameters	IgE	IgG4
Class-switch by	IL-4, IL-13 [52]	IL-4, IL-13 [52], IL-10
Enhanced secretion by	IL-5, IL-6, IL-7, IL-9 & IL-13 [52,55,57]	IL-10 [56], IL-21 [58,59,60]
Surface receptor binding	FcγR on mast cells and basophils	Low binding to FcγR on immune cells [51,66,67]
Precipitating immune complexes formation	(+)	(-) [61,62,66,67]
Complement activation	(+)	(-) [62,66,67]
Unique immunological effects	Allergic reaction	Anti-allergen antibody [51,53,54,64]
Therapeutic application	Anti-cancer IgE antibody [54]	Non-inflammatory monoclonal antibody [57,62,63,64,65,73]

**Table 5:** Odd immunological properties of IgG4 antibodies in patients with IgG4-RD.

IgG4 antibodies undergo a process of “Fab–arm exchange” to become half-antibodies with monovalency incapable of C1q activation and with low binding affinity to FcγRII and FcγRIII resulting in non-inflammatory property [51,62,67,68,69,70,71,72,73]
Anti-allergic effect by attenuation of Th2 cytokine-mediated inflammation and immunosuppression [51,53,54,63,72]
Exhibition of rheumatoid factor-like activity by Fc–Fc aggregation to resume activating complements [51,75]
IgG4 obtained from IgG4-RD subjects binds to normal epithelial cells of pancreato–hepatobiliary tissues and salivary glands in vitro [49]
Pathologic effects in certain autoimmune diseases including pemphigus foliaceus, muscle-specific kinase myasthenia gravis (MuSK MG) and idiopathic membranous nephropathy [76,77,78,79,80]
Complement activation and hypocomplementemia in IgG4-RD with unique-pattern glycosylation [81,82,83,84,85,86]

## Abbreviations

AID	activation-induced cytidine deaminase
AIP	autoimmune pancreatitis
AIT	allergen-induced immunotherapy
APC	antigen presenting cell
APRIL	a proliferation-inducing ligand
BAFF B	B lymphocyte-activating factor of tumor necrosis factor family
Bφ	basophil
Breg	regulatory B lymphocyte
C	complement component
CA	pancreatic carbonic anhydrase
CCL	C-C chemokine motif ligand
CD	cluster of differentiation
CP	chronic pancreatitis
CTGF	connective tissue growth factor
CXCL	C-X-C chemokine motif ligand
CXCR	C-X-C chemokine receptor
DAMP	damage associated molecular pattern
EC	endothelial cell
Eφ	eosinophil
FB	fibroblast
FcγR	Immunoglobulin G fragment C gamma receptor
FGFBP2	fibroblast growth factor binding protein 2
FliC	flagellin
GC	germinal center
GWAS	genome-wide association study
HLA	human leukocyte antigen
*H. pylori*	*Helicobacter pylori*
IgG4-RD	IgG4-related disease
IFN	interferon
IL	interleukin
IRF-7	interferon regulatory factor-7
K	lysine
LF	lactoferrin
LOXL2	lysyl oxidase-like 2
MAMP	microbe-associated molecular pattern
mCRP	monomeric C-reactive protein
MFB	myofibroblast
MMP	matrix metalloproteinase
MST	mammalian STE20-like protein kinase
Mφ	macrophage
NLR	nucleotide-binding oligomerization domain like receptor
P	proline
PAMP	pathogen-associated molecular pattern
PBMC	peripheral blood mononuclear cell
PBP	plasminogen-binding protein
*p*DC	plasmacytoid dendritic cell
PDGF	platelet-derived growth factor
PDGFB	platelet-derived growth factor B
PMN	polymorphonuclear neutrophil
Q	glutamine
R	arginine
RT-PCR	reverse transcriptase assisted polymerase chain reaction
S	serine
SIPS	systemic IgG4-related plasmacytic syndrome
SLAMF7	signaling lymphocytic activation molecule F7
SLE	systemic lupus erythematosus
SNP	single nucleotide polymorphism
SPINK1	serine peptidase inhibitor, Kazal type 1
PST1	pancreatic secretary trypsin inhibitor-1
PRSS1	protease serine 1 (trypsin 1)
ST2	suppression of tumorigenicity 2
Tc	cytotoxic T lymphocyte
TCN1	transcobalamin 1
T_EM_	T cells with effector memory phenotype
T_fh_	follicular helper T cell
TGF	transforming growth factor
Th	helper T lymphocyte
TIMP	tissue inhibitor of matrix metalloproteinase
TLR	Toll-like receptor
Treg	regulatory T lymphocyte
UBR2	ubiquitin–protein ligase E3 component n-region 2
